# Does Current United Kingdom Childhood Cancer Research Address the James Lind Alliance Children's Cancer Priority Setting Partnership Priorities? A Grant Mapping Study

**DOI:** 10.1111/hex.70845

**Published:** 2026-08-26

**Authors:** Alison Bish, Faith Gibson, Alexandra Brownsdon, Julia Chisholm, Jenny Harris, Rachel Hollis, Bob Phillips, Susie Aldiss

**Affiliations:** ^1^ School of Health Sciences University of Surrey Guildford UK; ^2^ Centre for Outcomes and Experience Research in Children's Health, Illness and Disability (ORCHID) Great Ormond Street Hospital for Children NHS Foundation Trust London UK; ^3^ Patient representative Exeter Devon UK; ^4^ The Royal Marsden NHS Foundation Trust Sutton UK; ^5^ Institute of Cancer Research Sutton UK; ^6^ Rachel Hollis Leeds Children's Hospital Leeds UK; ^7^ University of York York UK

**Keywords:** cancer, children, James Lind Alliance, priority setting partnership, research funding

## Abstract

**Introduction:**

The James Lind Alliance Children's Cancer Priority Setting Partnership identified 23 key research topics, as defined by children, survivors, families, carers, and professionals. Funders have expressed commitment to ensuring all priorities are addressed. The aim of our current project was to map UK‐funded childhood cancer research against the 23 priorities to highlight gaps and inform future funding, policy and research directions.

**Methods:**

A retrospective grant mapping study. Potential UK funders of childhood cancer research were contacted for details of projects funded January 2020–July 2025. Project abstracts were reviewed to map study aims to one or more of the 23 priorities. Total funding, co‐funders, type of cancer and study design were recorded.

**Results:**

Of 63 funders contacted, nine declined to participate, eight gave no response and 19 were ineligible as they had not funded relevant work in the study timeframe. Twenty‐seven funders provided data and three had online searchable databases, allowing direct data extraction: 452 studies were mapped. Studies covered all major childhood cancers; 75% were pre‐clinical in design. Total funding awarded was nearly £113 million with 39 co‐funded studies. Priority 1: *Developing effective and kinder treatments* received most research focus: with 365 studies and £94.2 million funding. Other well‐addressed priorities were Priority 2: *Why children develop cancer and can it be prevented*: with 73 studies and £16.6 million funding; and Priority 5: *Reasons for relapse* − 44 studies and £9.6 million. Five priorities received no funding, including Priority 6: *Improving hospital experience*, which was the top priority identified by children.

**Conclusion:**

While treatment research is well supported, some areas that matter to children, survivors, families and health professionals are overlooked. Funders and researchers should target underrepresented priorities. Policy makers can use findings to inform delivery of the National Cancer Plan. Patients and families can raise awareness of underfunded areas and influence funding decisions.

**Patient or Public Contribution:**

This mapping project stemmed from a James Lind Alliance Priority Setting Partnership that had at its core an emphasis on patient participation. Our project team included a survivor of childhood cancer.

## 1 Introduction

Within the United Kingdom (UK), between 1997 and 2016 around 33,000 children (aged 0‐14 years) were diagnosed with cancer, an average of 1,645 diagnoses per year [1]. Although cancer in children is rare, it is the most common cause of death in children in the UK [1].

Childhood cancer comes with a substantial burden; treatments can extend over several months to a few years and are associated with acute toxicities and long‐term health consequences. Children experience a variety of physical, psychological and social impacts relating to the disease, invasive procedures and treatments [2]. Welcome improvements in survival rates [3], with over 80% of children now surviving at least 5 years, also mean that an increasing number of people are living with the long‐term sequelae of cancer and its treatment. It is therefore vital that research addresses the issues which are most important to those affected by childhood cancer ‐ the children themselves and adult survivors of childhood cancer, their families and carers and the professionals who diagnose, treat and care for them. Ensuring alignment with their priorities will increase the chances of research being used and being most beneficial to all those affected.

**Figure 1:**
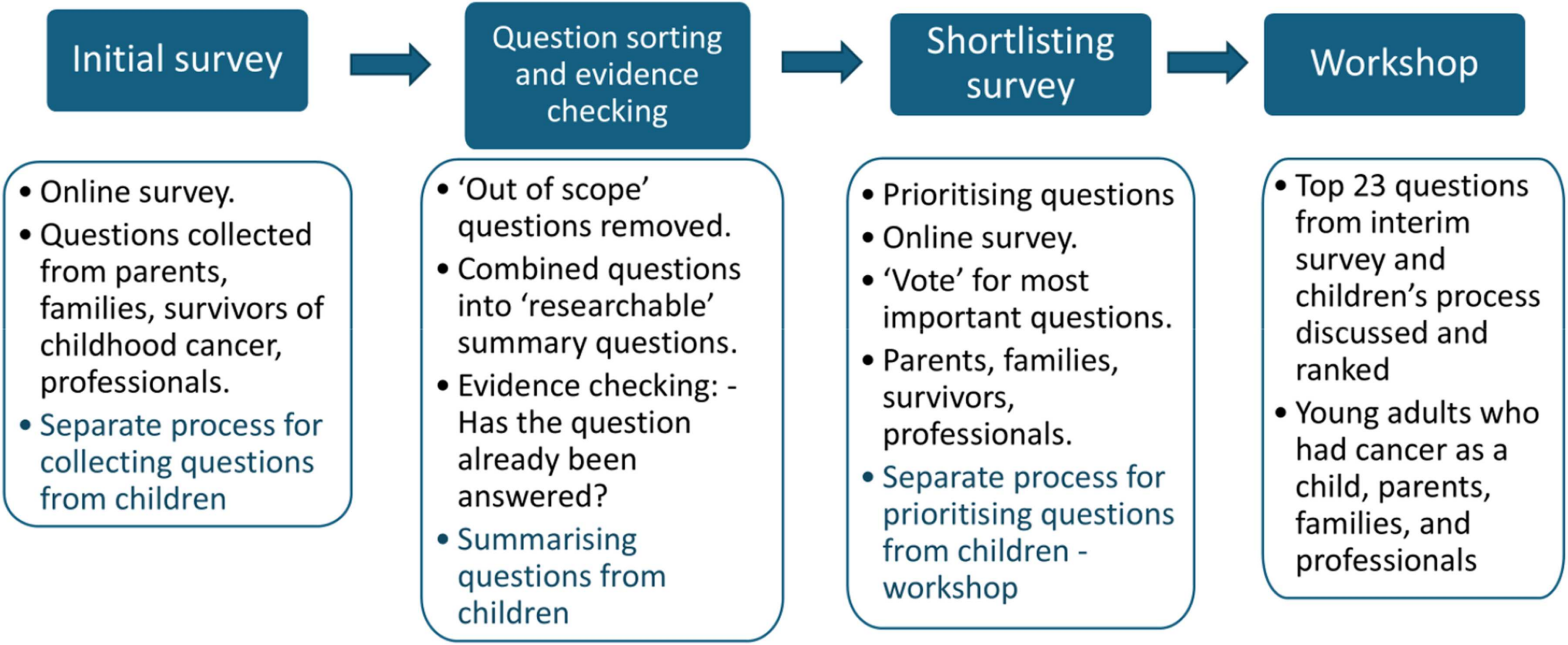
James Lind Alliance Priority Setting Partnership process.

In 2019, CCLG: The Children & Young People's Cancer Association and The Little Princess Trust partnered with the James Lind Alliance on the Children's Cancer Priority Setting Partnership (PSP). The aim was to identify the most important research questions in childhood cancer, as defined by children, survivors, families, carers, and professionals [4]. A systematic approach to priority setting (Figure 1), was followed which concluded with 23 questions discussed at a final workshop at which the Top 10 priorities were decided. These questions included the Top 5 identified by children and young people [5], alongside those prioritised by adult survivors of childhood cancer, families/carers, and professionals.

Recognising that work does not stop after the Top 10 is made available [6], the project team held a meeting with funders of children's cancer research following the priority setting exercise. Representatives from 16 cancer research funding organisations attended. The aim was to explore whether funders believed that the priorities were currently being addressed by their organisation and to gather views on what should be funded. Presentations at the meeting included perspectives from a parent, a survivor of childhood cancer, a professional and a funder of the PSP. Notably, during a group exercise, it was clear that funders’ perceptions of what their organisation was currently funding was largely aligned with what they believed should be funded. This raised the question ‐ do these funders think that the priorities which they do not fund are being funded from elsewhere, or were there simply other higher priorities they thought more important than those identified by stakeholders in the PSP? The study team have previously reflected on how far research priority setting exercises actually influence what research is undertaken: a little, a lot, or not at all? [7]. It was encouraging that in the final stage of this workshop a conclusion was reached that it was essential to understand much more about what is funded, to influence the research agenda in children's cancer care.

The agreed action from the meeting was this mapping study, reflecting a shared commitment to ensuring all priorities are addressed in any future research agenda. The aim was to highlight alignment or gaps with stakeholder priorities and to identify efficient use of resources.

Mapping projects like the present one are still relatively uncommon, even though James Lind Alliance PSPs are intended to guide research towards areas of unmet need. This makes studies which systematically identify those areas of unmet need especially important. Previous work has been carried out in the areas of palliative care/end of life [8, 9] and myalgic encephalomyelitis/chronic fatigue syndrome [10]. These projects found some alignment, but overall, only limited evidence that current research reflected the priorities identified in their PSPs. Others have developed an interactive online tool to help researchers identify overarching themes from completed PSPs in their specific health area, with the aim of enabling collaborative research to increase impact and make best use of limited research funds [11]. Around 5% of the themes available in the online tool involve cancer, although none specifically relate to children's cancer research.

## 2 Methods

This was a retrospective grant mapping study. The study aimed to systematically map current funding of childhood cancer research in the UK against the 23 priorities identified by the James Lind Alliance PSP.

The specific research objectives were to identify:

- Number of projects planned, ongoing, or completed per priority area.
- Amount of funding awarded per priority area.
- Number of funders addressing each priority area.
- Proportion of projects supported through co‐funding arrangements.
- Types of childhood cancers included in the funded studies.
- Methodologies used in the research.
- Extent of Patient and Public Involvement (PPI).

Funders were identified through multiple sources, including project partners in the Children's Cancer PSP, systematic review of cancer research organisations and recommendations from the project team and steering group.

All potential UK funders of childhood cancer research were contacted to determine their involvement in funding relevant research. Those confirming that they had funded relevant research were invited to provide details of projects funded between January 2020 and July 2025. This timeframe was chosen as it aligns with the launch of the Children's Cancer PSP survey in 2020 which aimed to collect topics for future research to focus on. Funders were contacted directly to ensure the most current and comprehensive dataset, including projects approved but not yet initiated. Up to two reminders were sent as needed. The first reminder was sent approximately 3 weeks after initial contact, with the second reminder up to a month later.

Mapping of individual studies was adapted from the grant mapping methodology used successfully by Marie Curie for the Palliative and End of Life Care PSP [9]. Participating funders provided details of studies that focussed on children with cancer or survivors diagnosed before age 16. Data requested included: project title, abstract, amount awarded and co‐funding details. For funders with open‐access searchable databases, relevant data were extracted directly.

Data were managed within Microsoft Excel. Titles and abstracts were screened against inclusion/exclusion criteria (see Table 1). Duplicate entries were removed from the dataset. Eligible abstracts were reviewed in detail to map study aims to one or more of the 23 PSP priorities.

**Table 1:** Study inclusion and exclusion criteria.

*Inclusion criteria*	*Exclusion criteria*
Funded between January 2020 and July 2025	Lacked an abstract, preventing mapping
Focussed on cancers with a prevalence > 1% among childhood cancers	Were infrastructure‐only grants (e.g. core funding, registry database setup, staffing costs)
For projects with human participants, included children, young people, or survivors (diagnosed under age 16)	
Were hosted by a UK‐based institution	
For personal awards (e.g., studentships, fellowships) that these had a defined project aim and monetary value	

The relationship between study aims and priorities was mapped as either:

- Direct: The primary aim addresses a PSP priority;
- Indirect: A secondary aim aligns with a PSP priority;
- No relationship.

Studies could be mapped to multiple priorities. Mapping was conducted by one researcher (AB), with a random 20% sample independently coded by a second researcher (SA). A rate of 20% was selected because the team considered this allowed sufficient coverage of the data to identify and discuss coding discrepancies and refine interpretations while remaining manageable within the study timeframe and resources. Microsoft Excel was used to generate a list of random numbers which corresponded to study identifiers. Discrepancies were resolved through discussion to reach consensus. Any uncertainties about inclusion were reviewed by the core study team (AB, SA, FG).

Descriptive statistics were used to summarise data.

## 3 Results

### 3.1 Response Rates and Funder Participation

After examining research strategies and websites of potential funders of children's cancer research it was likely that 66 funders may have funded standalone projects in the past 5 years. Three funders had searchable databases and therefore 63 funders were contacted directly. Responses were received from 54 (86%) of the 63 funders. No response was received from nine funders after three contact attempts, two were children's cancer charities and one was a cancer charity for all ages, the remaining six organisations were either focused on children's general health (n = 2) or all ages general health (n = 4). Nineteen funders confirmed that they had not funded research into children's cancer in the last 5 years and so were ineligible to participate. Eight declined to participate; reasons given for declining included:

- 3 lacking capacity or time
- 2 unable to share the data although they felt they had funded relevant work
- 1 ‘did not hold the data required’
- 1 ‘could not provide the information'
- 1 ‘could not take part'

Comprehensive coverage of the research landscape for childhood cancer within the last 5 years was achieved. Twenty‐seven funders provided data and databases were searched for a further three funders. Projects from these 30 funders were entered into the project database for mapping.

**Figure 2:**
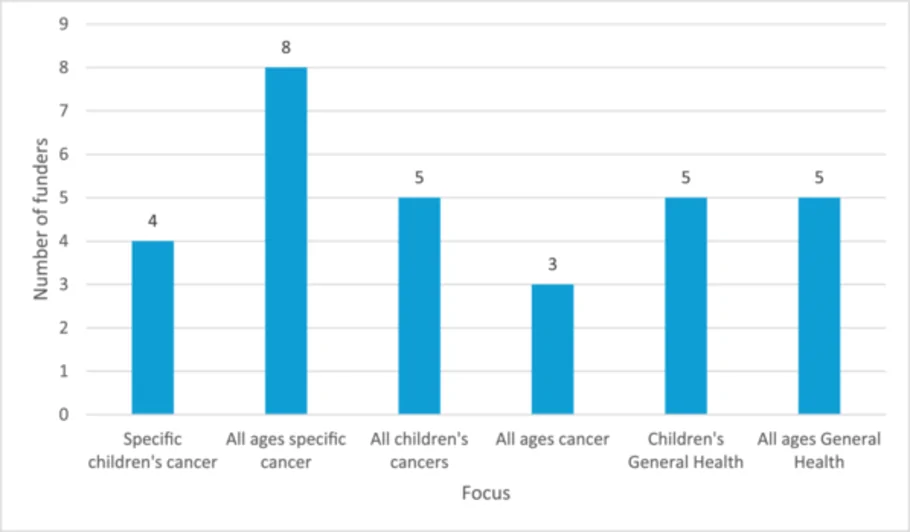
Focus of the participating funders.

Figure 2 shows the breakdown of the focus of the 30 funders included.

### 3.2 Funding Landscape and Research Characteristics

A total of 452 studies from the 30 funders were included; 253 (56%) had been completed and 199 (44%) were ongoing. Total funding for these 452 studies was £112,947,386 (median: £124,999; IQR £50,008–£249,856). Individual studies received between £2,520 and £4.17 million in funding, with most falling between £10,000 and £1 million. Thirty‐nine studies were co‐funded, with a total co‐funder contribution of £8,663,406.

The main childhood cancer types were covered by the studies, with around three quarters focussed on either leukaemia (27%), brain and spinal tumours (20%), neuroblastoma (13%), bone tumours (11%), or soft tissue sarcoma (7%) [1].

Nearly three quarters of funded studies were pre‐clinical, 75%, *n* = 337 (e.g. developing immunotherapy treatments; DNA profiling; examining drug resistance; repurposing existing drugs; examining tumour development; oncogenesis); 10% observational (*n* = 46) (e.g. cohort studies, cross‐sectional studies, qualitative research, machine learning), 8% clinical trials (*n* = 36), 2% reviews (*n* = 11); 2% intervention development (*n* = 10) and 1% feasibility studies (*n* = 5). The remaining studies (*n* = 7) were a mix of observational and pre‐clinical (*n* = 2); observational and review (*n* = 2); observational and health economics (*n* = 1); health economics (*n* = 1); implementation (*n* = 1).

Few studies mentioned involvement of patients or their families in their design. Patient and public involvement (PPI) was referred to in only 7% of the 452 study abstracts. Among those studies where children, teenagers, survivors, and families were participants (excluding pre‐clinical research) this increased to approximately 25%. However, it was felt that we could not reliably extract this data as many abstracts lacked detailed descriptions of methodology or engagement activities and therefore it was unclear if no PPI occurred in the study or whether it was simply underreported.

### 3.3 Mapping the Priorities

Table 2 shows the results of the mapping of individual studies against the PSP priorities. Studies could be mapped against more than one priority. The category ‘no relationship' was not used as no studies were found that did not map to a priority. As per our protocol a random sample of 20% of projects were independently mapped and coded against the priorities by a second researcher. Agreement between the two researchers undertaking the mapping was 88%.

**Table 2:** Priorities ranked in order reflecting number of studies which address this as their main or secondary aim, with additional details on funding awarded.

Original PSP priority number	Priority question	Number of studies where this priority is main aim of study (direct link)	Number of studies where this priority is secondary aim of study (indirect link)	Total funding (number of funders)
1	Can we find effective and kinder (less burdensome, more tolerable, with fewer short and long‐term effects) treatments for children with cancer, including relapsed cancer?	289	76	£94,197,669 (*n* = 29)
2^a^	Why do children develop cancer (including the role that genetics plays) and could it be prevented?	63	10	£16,570,910 (*n* = 19)
5	Why do children relapse, how can it be prevented, and what are the best ways to identify relapse earlier?	36	8	£9,639,086 (*n* = 13)
12	What are the best ways to reduce, predict and manage the side‐effects of treatment for children (including life threatening side‐effects)?	15	1	£5,312,657 (*n* = 7)
15	How common are the different long‐term effects of childhood cancer treatment, how do they change across the lifespan, can we predict them and how can they best be prevented, detected and/or treated?	14	0	£2,373,098 (*n* = 6)
8	What impact does cancer and treatment have on the lives of children and families after treatment, and in the long‐term; what are the best ways to help them to overcome these impacts to thrive and not just survive?	12	2	£1,810,679 (*n* = 7)
3	Are the psychological, practical, and financial support needs of children with cancer, survivors, and their families being met during treatment and beyond? How can access to this support be improved and what further support would they like?	7	1	£744,794 (*n* = 5)
4^a^	How can we speed up the process of getting diagnosed and starting treatment in the right place?	6	9	£2,558,098 (*n* = 11)
7	What are the best ways to ensure children and families get and understand the information they need, in order to make informed decisions, around the time of diagnosis, during treatment, at the end of treatment and after treatment has finished?	6	1	£980,363 (*n* = 4)
19	What fertility preservation options work best for children and teenagers with cancer?	3	1	£1,658,251 (*n* = 3)
11^a^	What are the best ways to provide emotional support for children and their families 1) around the time of diagnosis, 2) during treatment and 3) after treatment (including survivors who are now adults)?	3	0	£366,682 (*n* = 3)
10	What is the relationship between chronic fatigue syndrome, fibromyalgia, chronic pain and treatment for childhood cancer? (Fibromyalgia is a long‐term condition that causes pain all over the body.)	2	6	£1,968,352 (*n* = 2)
21	What are children's and survivors' experiences of the side‐effects and long‐term effects of cancer treatment?	2	3	£775,391 (*n* = 3)
9^a^	How can we make more accessible treatments that are closer to home, in shared care hospitals?	2	1	£204,959 (*n* = 2)
17	During and after treatment, what issues prevent or encourage physical activity, which interventions are most effective and what should be measured to assess effectiveness?	2	0	£100,438 (*n* = 2)
18	What are the best ways of making sure people who had cancer as a child receive the information they need about the long‐term effects of cancer and treatment?	2	0	£505,559 (*n* = 2)
23	What are the best ways to support children as they get older, and their needs change, to understand and take responsibility for their health, and to live with the long‐term effects of cancer and treatment?	1	1	£169,897 (*n* = 2)
14	What is the psychological and social impact of cancer and treatment on children and their families during treatment and in the long‐term; what factors affect these impacts?	1	0	£56,186 (*n* = 1)
6^a^	How can we make being in hospital a better experience for children and young people? (like having better food, internet, toys, and open visiting so other family members can be more involved in the child's care)	0	0	£0
13	How can transition (moving) from child into adult services be improved for young people who had cancer as a child?	0	0	£0
16	What are the best ways to support the emotional wellbeing of professionals who care for children with cancer and their families?	0	0	£0
20	What are the long‐term effects of additional medications children with cancer may receive (such as antibiotics, pain killers, laxatives) and how can these effects be reduced?	0	0	£0
22	How can experiences of having a Hickman line be improved for children with cancer? (A Hickman line is a small tube which is inserted into a vein so that treatments can be given, and blood taken without the repeated need to access veins with a needle. The Hickman line can stay in place for several months.)	0	0	£0

*Note:* Studies could be mapped against more than one priority.

^a^These priorities are one of the Top 5 from children and young people in the James Lind Alliance PSP.

Results indicate that not all Top 10 priorities are attracting research attention, and amongst those that are, this is at varying levels of research intensity and funding amount.

Three priorities combined accounted for approximately 80% of total funding of children's cancer research in the UK within the last 5 years (Priority 1, 2 and 5).

It is clear that Priority 1 (*Can we find effective and kinder (less burdensome, more tolerable, with fewer short and long‐term effects) treatments for children with cancer, including relapsed cancer?*) overwhelmingly receives the most research focus and funding, being a main or secondary aim in 289 (64%) and 76 (17%) of projects, and receiving £94,197,669 in funding from 29 funders. The majority (*n* = 310, 85%) of studies addressing this priority are at the pre‐clinical stage, therefore with a longer trajectory to patient benefit. Leukaemia (*n* = 104, 28%) and brain and spinal tumours were most studied (*n* = 78, 21%), followed by neuroblastoma (*n* = 50, 14%) and bone tumours (*n* = 46, 13%).

Priority 2 (*Why do children develop cancer (including the role that genetics plays) and could it be prevented?)* was the second most addressed by studies, with 73 (16%) covering this priority as either a primary or secondary aim. It received £16,570,910 in funding from 19 funders. Almost all (*n* = 67, 92%) studies were at the pre‐clinical stage. Investigations into leukaemia development and prevention were the most common (*n* = 31, 42% of studies), followed by neuroblastoma (*n* = 8, 11%) and bone tumours (*n* = 7, 10%).

Priority 5 (*Why do children relapse, how can it be prevented, and what are the best ways to identify relapse earlier?*) is the third most covered priority. Forty‐four (9.7%) studies received £9,639,086 in funding from 13 funders. Nearly three‐quarters of the studies were focussed on leukaemia (*n* = 16, 36%), brain and spinal tumours (*n* = 10, 23%), or soft tissue sarcoma (*n* = 7, 16%). Nearly three quarters (*n* = 32, 73%) were pre‐clinical in design and six (14%) were observational studies.

Three priorities focussing on issues around short and long term side effects and impact of cancer and treatment along with one priority on timely diagnosis were the next most covered, but by far fewer studies than the three priorities discussed above. Priority 12 *(What are the best ways to reduce, predict and manage the side‐effects of treatment for children (including life threatening side‐effects?)* was addressed by 16 (3.5%) studies which were awarded £5,312,657 by seven funders. In contrast to the previous priorities, only 13% (*n* = 2) of these studies were pre‐clinical, whereas 38% (*n* = 6) were observational. Over a third (*n* = 6, 38%) focussed on all childhood cancers.

Priority 15 *(How common are the different long‐term effects of childhood cancer treatment, how do they change across the lifespan, can we predict them and how can they best be prevented, detected and/or treated?)* was addressed by 14 studies (3%) with £2,373,098 awarded by six funders. Half of the studies were observational (*n* = 7, 50%) and 43% (*n* = 6) included all cancers.

Priority 8 (*What impact does cancer and treatment have on the lives of children and families after treatment, and in the long‐term; what are the best ways to help them to overcome these impacts to thrive and not just survive?)* was also addressed by 14 studies (3%) receiving £1,810,679 in funding from seven funders. Nearly two‐thirds (*n* = 9, 64%) of the studies included all cancers, and 57% (*n* = 8) were observational in design.

Priority 4 (*How can we speed up the process of getting diagnosed and starting treatment in the right place?)* was addressed by 15 (3.3%) studies. This was one of the top five priorities identified by children and young people in their workshop [5]. However, nine of the 15 studies addressed this only as a secondary aim. These studies received £2,558,098 in funding from 11 funders. Nearly half (*n* = 7, 47%) of the studies focussed on brain or spinal tumours and 53% (*n* = 8) were pre‐clinical in design.

Eleven priorities were addressed by fewer than ten studies (Table 2).

### 3.4 Critical Gaps in Research and Funding

Critically, five priorities are not the focus of any of the 452 studies identified. One of these is in the Top 10, Priority 6, (*How can we make being in hospital a better experience for children and young people*), and was in fact the top priority identified by children and young people in their prioritisation workshop for the PSP [5]. The remaining four receiving no research attention are Priority 13 (*How can transition from child into adult services be improved for young people who had cancer as a child?);* Priority 16 (*What are the best ways to support the emotional wellbeing of professionals who care for children with cancer and their families?*); Priority 20 (*What are the long‐term effects of additional medications children with cancer may receive and how can these effects be reduced?);* and Priority 22 *(How can experiences of having a Hickman line be improved for children with cancer?)*.

## 4 Discussion

This comprehensive mapping of 452 UK funded childhood cancer studies (totalling almost £113 million) reveals a striking imbalance. While treatment related research received overwhelming support, patient‐centred priorities including those identified by children in the PSP, remain largely unfunded. Priority 6 (improving hospital experience) ranked top by children received zero funding. Five of the 23 priorities attracted no research funding. It was encouraging to see that all major childhood cancers received some research attention, with the most common types generally attracting proportionately greater focus. Given that most of the current research is at the pre‐clinical stage, the translation of findings into tangible patient benefit is likely to follow a longer trajectory. This highlights the urgency for policy solutions that streamline the research process and reinforce the need for all forms of research across priority areas, to ensure that research delivers meaningful improvements in the lives of children with cancer and their families.

Previous grant mapping exercises have also identified gaps in funding of patient‐centred priorities. Consistent with our findings, the original grant mapping exercise undertaken by Marie Curie identified several priority areas that received little or no research funding in similar areas, including information needs, communication, provision of care, and support for patients and families [9]. Although the Marie Curie updated grant mapping exercise in 2023 found that some funding had subsequently been allocated to the Top 11 identified priorities, important mismatches between funded studies and ranked importance by stakeholders remained; for example Priority 1 (addressing the best ways of providing palliative care outside of ‘working hours') was ranked ninth out of the Top 11 priorities for receiving direct funding [8]. Boddy et al. [12] compared funded studies with patient and family identified research priorities relating to type 1 diabetes; they report an absence of funding focusing on the psychosocial impacts of living with diabetes. The authors suggest that funded research may be neglecting questions about the everyday reality of living with diabetes [12].

Our findings suggest a need for targeted funding initiatives to address underrepresented priorities ‐ particularly those identified by children and young people themselves and those that receive little or no attention. The results of this project can help shape future policy decisions on children's cancer research funding. This can be achieved by engaging with the Children and Young People Cancer Taskforce to inform the implementation of the National Cancer Plan [13] and embed the priorities into strategic planning. It is important that health and social care researchers working within the field of children's cancer research are made aware of the priorities that currently receive little or no funding. This will help them develop relevant, competitive funding applications and avoid duplicating efforts in areas that are already well supported. Increasing awareness among patients and the public about underfunded research priorities could help drive advocacy efforts – encouraging charities, government bodies, and other stakeholders to direct funding towards these unmet needs and contributing to campaigns and raising awareness through social media channels.

Funders could diversify funding beyond pre‐clinical studies and support translational research to bridge the gap between pre‐clinical work and research with clinical and real‐world applications. It would be useful to expand beyond pre‐clinical studies to include observational, qualitative, intervention development, and implementation research and to explore mixed‐methods approaches to capture the complexity of lived experiences. This would be especially useful in addressing some of the underfunded and neglected James Lind Alliance PSP priorities. Increasing the range of methodologies used may also provide more opportunities for patients to participate in research studies where possible, especially those involving lived experience and survivorship issues.

### 4.1 Strengths and Limitations

A strength of the current study is that funders were contacted directly, and the use of searchable databases meant that completed, ongoing, and yet to start work could all be included – allowing for the most comprehensive overview to date of childhood cancer research funding in the UK. This is in contrast to some other mapping work which focus on published research only [9, 10]. In addition, there was high engagement from the funding community. However, whilst substantial efforts were made to include all relevant funders it is possible that smaller charities may have been missed. Some grants had a limited abstract which made mapping difficult and may not therefore fully represent the aims of the study. This highlights the need for researchers to supply informative abstracts and lay summaries for research awards. By excluding infrastructure spending and focussing solely on standalone projects, the study may have overlooked research conducted within established research centres ‐ for instance, fellowships or smaller studies led by senior researchers.

We were unable to fulfil one of our aims – to examine the extent of PPI involvement in funded studies as this was rarely reported in the abstracts reviewed. This does not necessarily mean that PPI involvement was lacking, the omission could be due to abstract word limitations. Involving children, young people, survivors and families in PPI activities including identifying priorities and planning how research is undertaken ensures that research is more relevant, accessible and meaningful [14, 15]. We recommend that funders mandate meaningful PPI in funded projects, and support researchers to engage with stakeholders effectively, in terms of time and money. Grant review panel members should ensure that PPI is embedded in research projects from the earliest stages. Researchers should actively involve children, young people, survivors, and families in their research designs and dissemination. To improve the visibility and recognition of these activities, PPI should also be clearly reported in research abstracts.

## 5 Conclusion

This mapping study has revealed a substantial misalignment between research funding and stakeholder priorities. We welcome the more recent National Institute for Health and Care Excellence ongoing call for James Lind Alliance PSP informed studies (https://www.nihr.ac.uk/nihr-james-lind-alliance-priority-setting-partnerships-rolling-call). This research should be used to guide policy around funding priorities. In the future it would be useful to establish shared databases to track research and how far it is aligned with James Lind Alliance priorities. A similar approach to this mapping study could be undertaken every 5 years to assess the funding landscape and continue to identify gaps.

## Author Contributions

**Alison Bish:** formal analysis, investigation, project administration, writing – original draft. **Faith Gibson:** conceptualisation, funding acquisition, methodology, formal analysis, project administration, writing – review and editing. **Alexandra Brownsdon:** funding acquisition, writing – review and editing. **Julia Chisholm:** funding acquisition, writing – review and editing. **Jenny Harris:** funding acquisition, writing – review and editing, formal analysis, visualisation. **Rachel Hollis:** writing – review and editing, funding acquisition. **Bob Phillips:** funding acquisition, writing – review and editing. **Susie Aldiss:** conceptualisation, funding acquisition, writing – review and editing, methodology, formal analysis, project administration.

## Conflicts of Interest

The authors declare no conflicts of interest.

## Data Availability

The data are not publicly available due to privacy or ethical restrictions. The final report, containing additional detail can be accessed here: https://www.cclg.org.uk/what-we-do/childrens-cancer-priority-setting-partnership/mapping-funding-childhood-cancer-research-priorities.
